# The Effect of In Concentration and Temperature on Dissolution and Precipitation in Sn–Bi Alloys

**DOI:** 10.3390/ma17174372

**Published:** 2024-09-04

**Authors:** Qichao Hao, Xinfu Tan, Qinfen Gu, Stuart D. McDonald, Kazuhiro Nogita

**Affiliations:** 1 Nihon Superior Centre for the Manufacture of Electronic Materials, School of Mechanical and Mining Engineering, The University of Queensland, St. Lucia, QLD 4072, Australia; q.hao@uq.edu.au (Q.H.); xin.tan@uq.edu.au (X.T.); s.mcdonald1@uq.edu.au (S.D.M.); 2 Australian Synchrotron, ANSTO, Clayton, VIC 3168, Australia; qinfeng@ansto.gov.au

**Keywords:** electronics packaging, low-temperature solder, Sn–Bi alloys, PXRD, DFT

## Abstract

Sn–Bi-based, low-temperature solder alloys are being developed to offer the electronics manufacturing industry a path to lower temperature processes. A critical challenge is the significant microstructural and lattice parameter changes that these alloys undergo at typical service temperatures, largely due to the variable solubility of Bi during the Sn phase. The influence of alloying additions in improving the performance of these alloys is the subject of much research. This study aims to enhance the understanding of how alloying with In influences these properties, which are crucial for improving the alloy’s reliability. Using in situ heating synchrotron powder X-ray diffraction (PXRD), we investigated the Sn–57 wt% Bi–xIn (x = 0, 0.2, 0.5, 1, 3 wt%) alloys during heating and cooling. Our findings reveal that In modifies the microstructure, promoting more homogeneous Bi distribution during thermal cycling. This study not only provides new insights into the dissolution and precipitation behaviour of Bi in Sn–Bi-based alloys, but also demonstrates the potential of In to improve the thermal stability of these alloys. These innovations contribute significantly to advancing the performance and reliability of Sn–Bi-based, low-temperature solder alloys.

## 1. Introduction

In the realm of electronics packaging, low-temperature soldering emerges as a critical process, aligned with the trend towards electronic device miniaturisation [1,2]. This technique offers substantial benefits, including diminished energy consumption and safeguarding electrical components against thermal damage, along with preventing mismatches in the Coefficient of Thermal Expansion (CTE) between the components and printed circuit boards. Following the introduction of the Directive on the Restriction of Hazardous Substances (RoHS) by the European Union [3,4,5,6], lead-free solder alloys from Sn–3.0 wt% Ag–0.5 wt% Cu (SAC), with a melting point of 220 °C, became the most common choice for reflow soldering [7]. There is research demonstrating the lowering of the melting point of SAC alloys with In additions and the melting point of SAC-based solders has been lowered to around 200 °C [8,9]. More generally, it is estimated that there is a 1.9 degree drop in the liquidus temperature per one weight percent addition of In [10]. As mentioned above, a new requirement has emerged for low-temperature solder alloys to meet the demand in terms of next-generation devices and solder alloys with a melting point under 200 °C [11,12]. Among various low-temperature solder alloys, such as Sn–In, Sn–Bi, Sn–Zn, and In–Bi, Sn–Bi has garnered significant interest due to its appealing eutectic temperature of 139 °C, its cost effectiveness, and its non-toxic characteristics [2,11,13,14]. However, there are still disadvantages associated with the Sn–Bi alloy, such as poor wettability and brittleness [2]. In order to improve the alloy’s performance, there has been a lot of research on adding ternary elements to Sn–Bi-based solder alloys, such as Ag, Cu, Sb, and In [15,16,17,18,19,20,21].

Reliability during thermal cycling is one of the most crucial factors for solder joints, as the solder undergoes continuous thermal cycling in operational electronic devices. Evidence suggests that In can enhance the reliability of Sn–Bi solder alloys during thermal cycling [22]. However, the exact mechanism remains unclear. Our previous studies have shown that Sn–Bi-based solder alloys are sensitive to temperature changes, primarily due to Bi dissolution into and precipitation during the Sn phase [17,23,24,25,26]. Based on our research, it is hypothesised that the enhanced thermal cycling reliability with the In addition, is due to the altered dissolution and precipitation behaviour of Bi during the Sn phase. This work aims to investigate the effect of In on the microstructure and crystal structure of Sn–Bi solder alloys under thermal cycling, to reveal the underlying material science in order to validate the hypothesis. This investigation will provide valuable insights for alloy design, leading to significant enhancements in the reliability and longevity of electronic devices.

The rapid dissolution and precipitation of Bi within the Sn–Bi–In alloy present significant challenges for analysis using traditional tools, such as scanning electron microscopy (SEM), lab-based X-ray diffraction (XRD), or energy dispersive X-ray spectroscopy (EDS), due to their limited spatial observation windows and high spatial resolution requirements. In situ heating synchrotron powder X-ray diffraction (PXRD), with its high-speed scanning and precise temperature control, emerges as a powerful alternative for examining the lattice parameters of materials under these conditions. By analysing the PXRD patterns and lattice parameters of Sn–57Bi–xIn alloys, we can accurately depict the dissolution and precipitation processes. Furthermore, the integration of SEM for microstructural analysis, cooling curve analysis of the thermal behaviour, CALPHAD (calculation of phase diagrams), and density functional theory (DFT) calculations for theoretical insights, enables a comprehensive understanding of the thermal changes in Sn–57Bi–xIn alloys. The objective of these experiments and simulations is to study the influence of the In concentration on the morphology of Sn–Bi–In alloys, as well as the dissolution and precipitation of Bi within the alloy during temperature variations. This knowledge could serve as a fundamental basis for improving the thermal behaviour of Sn–Bi-based solder alloys. This study offers significant insights into the third element’s influence on Sn–Bi based, low-temperature solder alloys, enhancing our scientific understanding of their thermal behaviour.

## 2. Materials and Methods

### 2.1. Sample Preparation

Sn–57Bi–xIn alloys (where x = 0, 0.2, 0.5, 1, and 3 compositions in wt% unless otherwise specified) were cast using pure Sn, Bi, and In ingots (the ingots, comprising 99.99% pure Sn and Bi sourced from Hayes Metals Pty, Australia, and 99.99% pure In obtained from Dowa Metals and Mining Co., Ltd., Tokey, Japan, were utilized in this study.). These compositions were chosen because the eutectic alloy Sn57Bi, known for its low melting point within the Sn–Bi system, has been extensively studied, and the gradual increase in the In content was intended to explore its effects on the alloy’s properties. The Sn, Bi, and In ingots were melted at 450 °C in an electric resistance furnace for 1 h, with frequent stirring to ensure the alloy’s homogeneity. Before casting in a preheated mould, the dross, the oxide, and the impurity-rich layer on the liquid metal surface, were carefully removed.

### 2.2. Material Characterisation

Cooling curve analysis was conducted by solidifying approximately 60 g of each alloy sample in a graphite cup. A thermocouple (sourced from RS Components Pty Limited, Smithfield, NSW, Australia) was inserted into the centre of the molten alloy and a data logger recorded the temperature changes as the samples cooled from 280 °C to below 50 °C, achieving full solidification. This process, performed through natural cooling in ambient air, is critical for understanding the solidification behaviour and properties of the alloys.

The microstructures of Sn–57Bi–xIn (x = 0, 0.2, 0.5, 1, and 3 wt%) alloys were analysed using SEM. Samples from the as-cast ingots were cold mounted in epoxy resin (sourced from Thermo Fisher Scientific, Scoresby, VIC, Australia), then sequentially ground with #320, #600, #1200, and #4000 silicon carbide paper, and polished to a final 0.25 µm finish (sanding and polishing disks are supplied by Struers, Champigny-sur-Marne, France). To enhance the electron conductivity for imaging, the polished samples were coated with a 10 nm gold layer. SEM images were acquired in backscatter electron (BSE) mode, using a Hitachi TM3030 SEM (sourced from Hitachi, Tokey, Japan), at an accelerating voltage of 15 kV, allowing detailed observation of the alloy microstructures.

**Figure 1:**
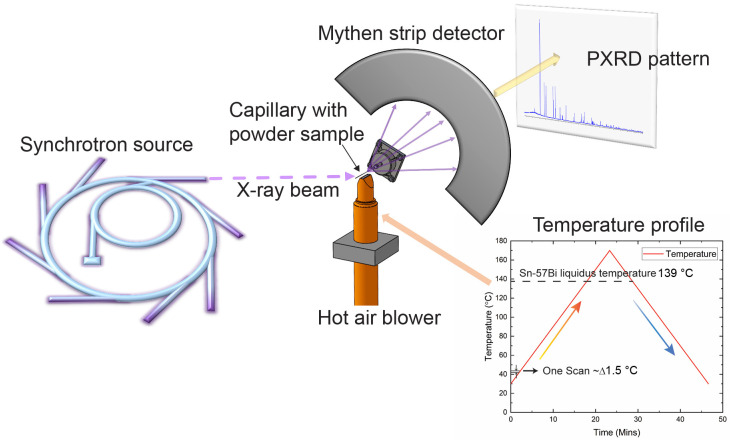
Schematic of the in situ synchrotron PXRD for Sn–57Bi–xIn (x = 0, 0.2, 0.5, 1, and 3 wt%) alloy samples.

The samples for the PXRD experiments were prepared by cutting the cast ingots with a saw. The resulting swarf was then crushed in an agate mortar into a fine powder for loading into capillaries (supplied by ProSciTech Pty Ltd., Kirwan, QLD, Australia), aimed at achieving a suitable particle size for the synchrotron PXRD experiments. These experiments were carried out at the powder diffraction beamline of the Australian Synchrotron (located at Melbourne, Australia). To mitigate X-ray attenuation by the high mass energy-absorbing Bi, the powder samples were diluted with ground quartz capillary powder (supplied by ProSciTech Pty Ltd., Australila). The resultant mixture was placed in a quartz capillary, with an internal diameter of 500 μm, and mounted on a rotary holder above a hot air blower (supplied by Australian Synchrotron, Clayton, VIC, Australia). The experiments were conducted under atmospheric conditions. The PXRD patterns were recorded during continuous heating from 30 °C to 170 °C at 6 °C/min under atmospheric pressure, with continuous cooling back to 30 °C upon reaching 170 °C, at the same temperature ramp rate (Figure 1). Continuous scanning at one position was employed, with each scan lasting 10 s, covering approximately 1.5 °C, thus providing a continuous reflection of the properties. Continuous scanning at one position means that the scanning has a gap at certain 2–theta positions. However, the peaks selected for comparison in this article avoid these positions to make the situation comparable. The 21 keV monochromatic incident beam, calibrated using a standard LaB_6_ sample (NIST660b) for precise wavelength determination at room temperature, resulted in a wavelength of 0.5922 Å. The heating rate was controlled to 6 °C/min. The diffracted patterns were captured in transmission mode, using a Mythen-II strip detector, maintaining a consistent comparison of the peak positions. 

The phase identification and the lattice parameters of each phase were derived from the Rietveld refinement of the 2–theta range from 8° to 45°of each sample, using TOPAS Academic v6 (Bruker-AXS, Madison, WI, USA). The tetragonal βSn (PDF number: 00–004–0673, space group *I41*/*amd*, a = 5.831 Å, c = 3.182 Å) and trigonal Bi (PDF number: 00–044–1246, space group *R*$\bar{3}$*m*, a = 4.547 Å, c = 11.863 Å) structures were used as the starting structure for the Rietveld refinement. The weighted profile R-factor (RWP) of all the refinements was controlled below 10 to ensure a good fit between the experimental and refined data.

### 2.3. DFT Calculation

Since the microstructure and crystal structure changes are significantly more substantial during the Sn phase for Sn–Bi-based solder alloys, DFT calculations were conducted using the Vienna ab initio simulation package (VASP) version 6.2.1 code to study the interactions among the Sn–In and Sn–Bi–In atoms during the Sn phase, at the Bi and In concentrations relevant to this study. The ion–electron interaction was described, utilising the projector-augmented wave (PAW) method. For estimating the exchange-correlation potential, we employed the generalised gradient approximation (GGA) within the Perdew–Burke–Ernzerhof (PBE) formulation. To accurately represent alloys with low concentrations of Bi and In, a supercell approach was adopted. Specifically, a supercell configuration consisting of 4 × 4 × 8 tetragonal β-Sn unit cells, corresponding to the powder diffraction file (PDF) number detailed in Section 2.2, was initialised with 512 Sn atoms. This structure served as the basis for our simulations and the models were constructed using the VESTA (Ver 3.5.8) software [27]. The computational settings were standardised across all the simulations, with a plane-wave cutoff energy of 500 eV. Given the substantial size of the structure, exceeding 23 Å along each axis, a minimal k-point mesh of 1 × 1 × 1 was deemed sufficient for our calculations. 

To assess the stability of each configuration, we calculated the formation energies per atom for structures resulting from the substitution of y Bi atoms and z In atoms into the Sn supercell, where y = 0, 12, 24, and 32, and z = 0, 1, 2, and 4. These calculations were performed according to the following equation:

(1) $$\Delta E_{f}=\frac{1}{512}(E\left[{Sn}_{512-y-z}{Bi}_{y}{In}_{z}\right]+\left(y+z\right)E\left[Sn\right]–E\left[{Sn}_{512}\right]–yE\left[Bi\right]–zE\left[In\right])$$

where *ΔE_f_* represents the formation energy per atom. Here, *E*[Sn_512-y-z_Bi_y_In_z_] and *E*[Sn_512_] denote the total energy of the Sn–Bi–In supercell and the pure Sn supercell, respectively. The terms *E*[Sn], *E*[Bi], and *E*[In] correspond to the total energy per atom for the tetragonal Sn, trigonal Bi, and tetragonal In, respectively. The unit cells for these calculations were chosen based on their crystal structures, namely tetragonal βSn and trigonal Bi, with their respective PDF numbers provided in Section 2.2, and tetragonal In, which is specified by the PDF number 00-005-0642, with a space group of I4/mmm, and lattice parameters of a = 3.252 Å and c = 4.9466 Å.

## 3. Results and Discussion

### 3.1. Microstructure

**Figure 2:**
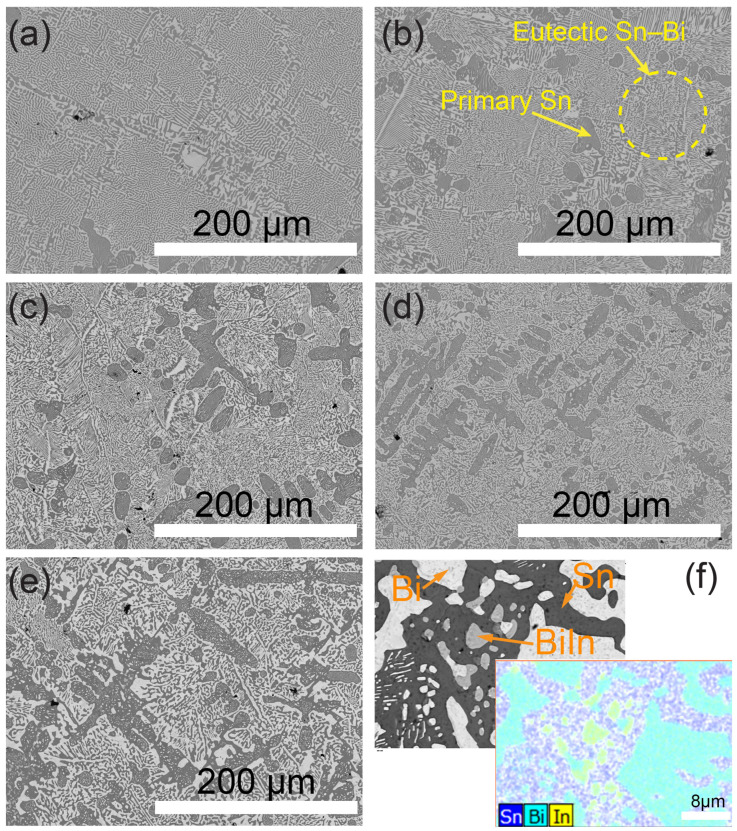
SEM and EDS images of the Sn–57Bi–xIn (x = 0, 0.2, 0.5, 1, and 3 wt%) alloys: (**a**) Sn–57Bi, (**b**) Sn–57Bi–0.2In, (**c**) Sn–57Bi–0.5In, (**d**) Sn–57Bi–1In, (**e**) Sn–57Bi–3In, and (**f**) EDS mapping of Sn–57Bi–3In.

Figure 2 illustrates the microstructure of the Sn–57Bi–xIn alloys (x = 0, 0.2, 0.5, 1, and 3 wt%). The Sn–57Bi alloy exhibits a eutectic structure, with a minor presence in terms of the primary Sn phase. As the concentration of In increases, noticeable changes occur in the microstructural morphology. Initially, the addition of In leads to a significant increase in the amount of primary Sn (see Figure S1), alongside a refinement of the eutectic structure for concentrations of In below 1%. The percentage of primary Sn vs. wt% In was quantified by analysing the images in Figure 2a–e with ImageJ software. In regard to the Sn–57Bi–3In alloy, the eutectic phases become coarser and the size of the Sn lamellar broadens. The refinement of the eutectic structure and the coarsening when the In concentration increased aligns with the findings by Wu, X., et al., who studied the influence of In on Sn–40Bi–xIn alloys [21]. BiIn intermetallic compounds, appearing as a grey phase between the dark coloured Sn and bright coloured Bi, are exclusively observed in the Sn57Bi3In sample. This observation aligns with the elemental distribution map in Figure 2.

These findings are consistent with the research conducted by Chen, X., et al. [18], which reported the absence of BiIn intermetallic compounds in alloys with 1 wt% and 2 wt%, in addition to the eutectic Sn–Bi matrix. BiIn intermetallic compounds were only identified in concentrations exceeding 4 wt% In, a conclusion supported by X-ray diffraction (XRD) analyses [18]. However, the referenced study did not report on the BiIn phases in terms of the Sn–58Bi–3In samples, nor were they detected in XRD scans, possibly due to the slight compositional differences compared to our samples. Our results align with the Thermo-Calc predictions (see Supplementary Figure S2), indicating the formation of BiIn intermetallic compounds at around 75 °C during the solidification of Sn–57Bi–3In, unlike the Sn–57Bi–0.5In and Sn–57Bi–1In variants.

### 3.2. Cooling Curve Analysis

**Figure 3:**
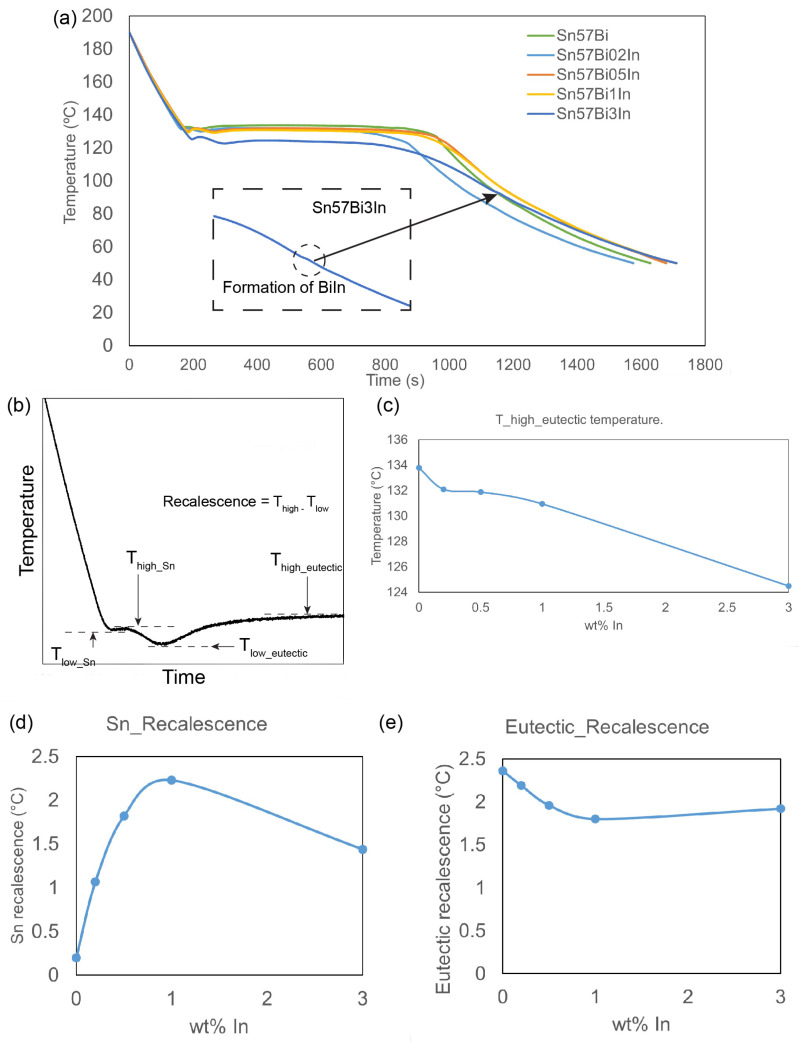
(**a**) Cooling curve during solidification of Sn–57Bi–xIn (x = 0,0.2, 0.5, 1, and 3 wt%) alloys, (**b**) definition of recalescence, (**c**) eutectic temperature, recalescence during the solidification, (**d**) primary Sn, and (**e**) eutectic Sn–Bi.

The calibration of the thermocouples during the cooling analysis was achieved using the melting point of a pure Sn sample. Figure 3a presents the cooling curves for the Sn–57Bi–xIn samples. The analysis of these curves, in conjunction with the microstructural observations from Figure 2 and the solidification sequence depicted in Supplementary Figure S2, allows for a comprehensive understanding of the cooling process [17,28]. Based on the microstructure in Figure 2 and the solidification process in Figure S2, it can be concluded that during the cooling process, microstructural evolution occurs through the formation of primary βSn dendrites, followed by eutectic Sn–Bi phases and, in samples with 3 wt% In, the formation of small quantities of the BiIn phase. 

Given that the composition of the Sn–57Bi–xIn alloys are close to eutectic alloys, the eutectic phases predominate within these alloys. The temperature changes observed during cooling serve as indicators of phase formation: sudden discontinuities in the slope of the cooling curve signals the onset of each phase’s formation, while the large temperature plateaus denote periods of eutectic phase formation. Notably, the incorporation of In into the alloy leads to a reduction in the eutectic temperature, which decreases from 133.79 °C in the Sn–57Bi alloy to 124.49 °C in the Sn–57Bi–3In alloy, as shown in Figure 3c.

The recalescence [29], as defined and illustrated in Figure 3b, refers to the phenomenon where the temperature of an alloy increases during cooling, a result of the latent heat released as the material transitions from a liquid to a solid state. Figure 3d and Figure 3e depict the recalescence observed for the primary Sn phase and the eutectic phase, respectively. The observed increase in the recalescence for Sn, with the addition of In, suggests a more pronounced release of latent heat during the Sn phase solidification process, indicating increased difficulty in regard to nucleating the Sn phase. The initial decrease in eutectic recalescence (Figure 3e), observed with the incorporation of a minor quantity of In (up to 1 wt%), suggests that certain concentrations of In contribute to the refinement of the eutectic structure.

### 3.3. In Situ Synchrotron PXRD 

To investigate the effects of In on the Sn–Bi alloys and simulate the thermal cycling process, the samples underwent heating and cooling continuously during the PXRD scanning. Prior research has established that the most pronounced transformations within Sn–Bi-based alloys during such thermal cycling are the dissolution and re-precipitation of Bi within the Sn phase [25]. Consequently, our analysis during thermal cycling was primarily concentrated on monitoring the βSn peaks, as these provide the most direct insight into the structural changes taking place within the alloy.

#### 3.3.1. PXRD Patterns at Room Temperature

**Figure 4:**
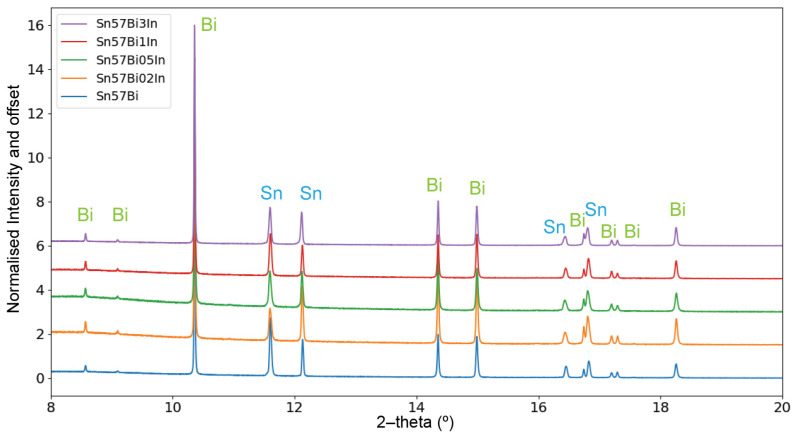
Normalised PXRD patterns for Sn–57Bi–xIn (x = 0, 0.2, 0.5, 1, and 3 wt%) alloys at room temperature.

Figure 4 displays the normalised PXRD patterns of Sn–57Bi–xIn alloys (x = 0, 0.2, 0.5, 1, and 3 wt%) measured at room temperature. The patterns reveal the presence of the βSn and Bi phases across all the samples. Notably, in the Sn–57Bi–3In alloy, the BiIn intermetallic phase was not discernible, potentially due to the small quantity of the phase in combination with the dilution with powdered quartz and the limited duration of the scan, which may have been insufficient for the BiIn peak to emerge distinctly. 

From the 10 s room temperature scans, it is discerned that the Sn peaks have a good signal-to-noise ratio, suitable for Rietveld refinement and the in situ investigation of the alloys during continuous heating and cooling. Particularly, the prominent Sn (200) peak, found between 11° to 12° 2–theta, was chosen for detailed observation of the Sn peak transformations throughout the heating and cooling process. This peak’s behaviour during heating and cooling provides valuable insights into the Bi precipitation and dissolution phenomena.

#### 3.3.2. PXRD Patterns during Heating

**Figure 5:**
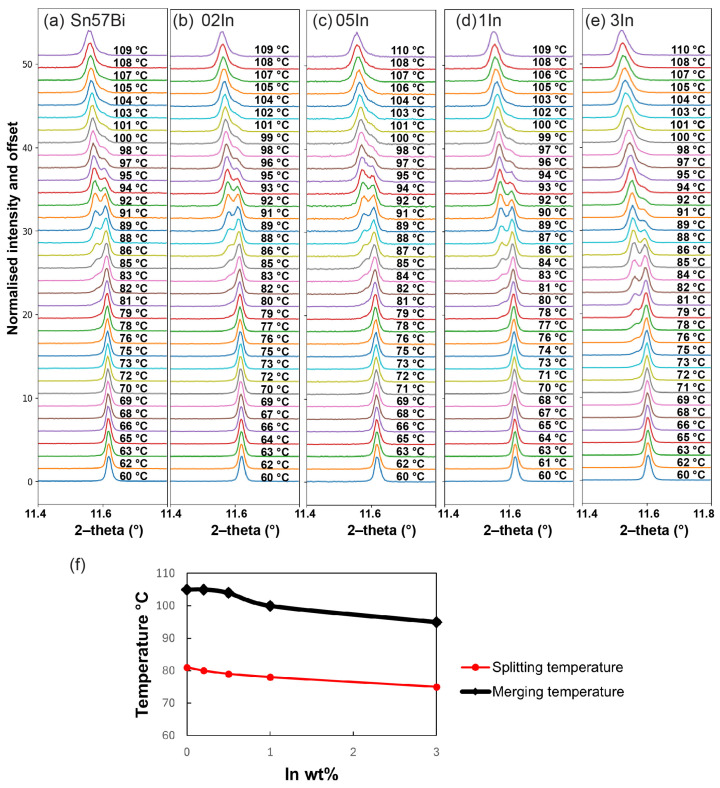
Normalised Sn (200) peak during heating for: (**a**) Sn–57Bi, (**b**) Sn–57Bi–0.2In, (**c**) Sn–57Bi–0.5In, (**d**) Sn–57Bi–1In, and (**e**) Sn–57Bi–3In, and (**f**) the splitting temperature and merging temperature for the Sn (200) peak.

During the heating process, Figure 5 demonstrates that for Sn–Bi–In alloys, the Sn (200) peak undergoes splitting at certain temperatures, which corresponds to the accelerated dissolution of Bi into the Sn matrix, creating a bimodal distribution of the concentration of Bi during the Sn phase. As the temperature increases, the Sn phase regains homogeneity, which causes the peaks to merge back into one. This temperature-dependent peak splitting and, subsequent, merging are markedly influenced by the addition of In, as seen in the progression from a lower temperature of 60 °C to around 110 °C, where the peaks have already coalesced.

**Figure 6:**
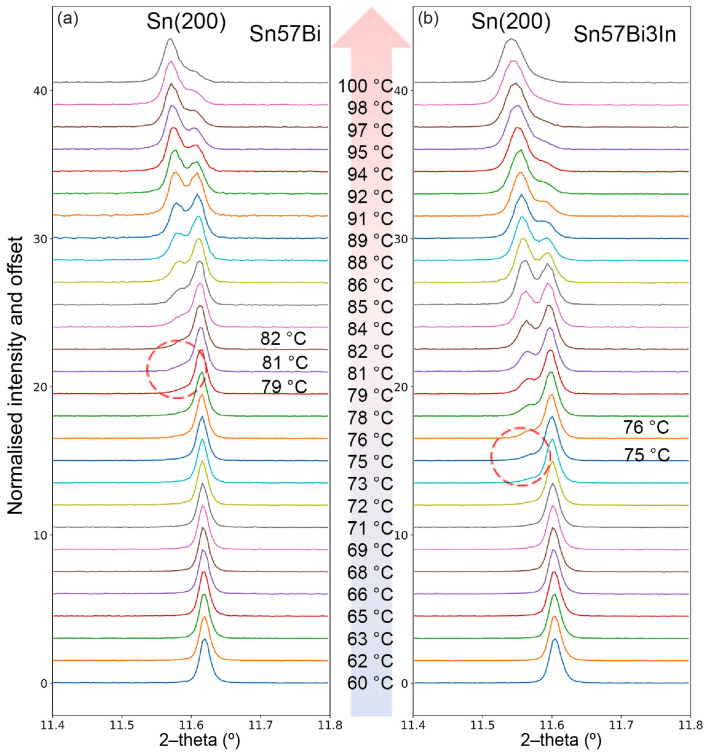
Normalised Sn (200) peak during heating for (**a**) Sn–57Bi and (**b**) Sn–57Bi–3In. Curves have been offset with temperature for clarity.

The addition of In modifies the temperature at which peak splitting initiates and where it concludes. While the peak splitting and merging temperatures for additions of up to 0.5 wt% In are quite similar and challenging to differentiate, the temperatures for both phenomena are noticeably reduced for the Sn–57Bi–1In and Sn–57Bi–3In alloys, as shown in Figure 5f. For a better understanding of this phenomenon, the evolution of Sn peaks in the Sn–57Bi and Sn–57Bi–3In alloys upon heating from 60 °C to 100 °C are selected for comparison in Figure 6.

At lower temperatures, an initial shift in the Sn peaks to lower 2–theta angles is observed for both alloys, indicative of an increase in the lattice parameters due to thermal expansion. Notably, during the heating process, a peak split occurs, signalling the onset of Bi dissolution into the Sn lattice, resulting in a non-uniform bimodal distribution of Bi within the Sn phase. This split manifests around 80 °C for Sn–57Bi and 75 °C for Sn–57Bi–3In, suggesting that the presence of In facilitates Bi dissolution at a lower temperature, likely contributed to by the dissolution of the BiIn phase in the Sn–57Bi–3In alloy, as shown in Figure 2f. As the Bi dissolution speed is high and not homogeneous at this stage, there exists two kinds of Sn phases with a rich Bi concentration and a lower Bi concentration. The peak corresponding to the Bi-rich Sn exhibits a higher lattice parameter, while the Bi-poor Sn shows a lower lattice parameter, leading to the observed peak splitting. As heating continues, the dissolution process progresses, resulting in the merging of the split peaks into a single peak, with a higher Bi concentration. The merging of peaks is completed at approximately 95 °C for Sn–57Bi–3In, whereas for Sn–57Bi, the peaks have not fully coalesced, even at 100 °C. 

This behaviour indicates that the In addition not only facilitates the homogenisation of the Sn phase at elevated temperatures, but could also potentially influence the thermal stability and solubility limits within the Sn–Bi system. The In addition causes the Sn peaks to merge at a lower temperature, suggesting that In may be affecting the temperature at which the Sn phase becomes homogenous. A homogenous phase at a lower temperature could mean that the material maintains its desired structure over a wider range of temperatures. However, the low splitting temperature is not ideal for the range of normal applications that apply within the range from room temperature to about 100 °C. The lowered splitting temperature shortens the temperature range over which the structure is homogeneous. However, in regard to the thermal cycling conditions, both heating and cooling should be considered to investigate the stability of the alloy. The cooling condition is discussed in Section 3.3.4. Also, the merging of the Sn peaks at different temperatures indicates that the solubility of Bi in the Sn lattice is affected by the presence of In. The peak merging means the alloy becomes more homogeneous and In promotes this phenomenon.

#### 3.3.3. Lattice Parameters of Sn during Heating

**Figure 7:**
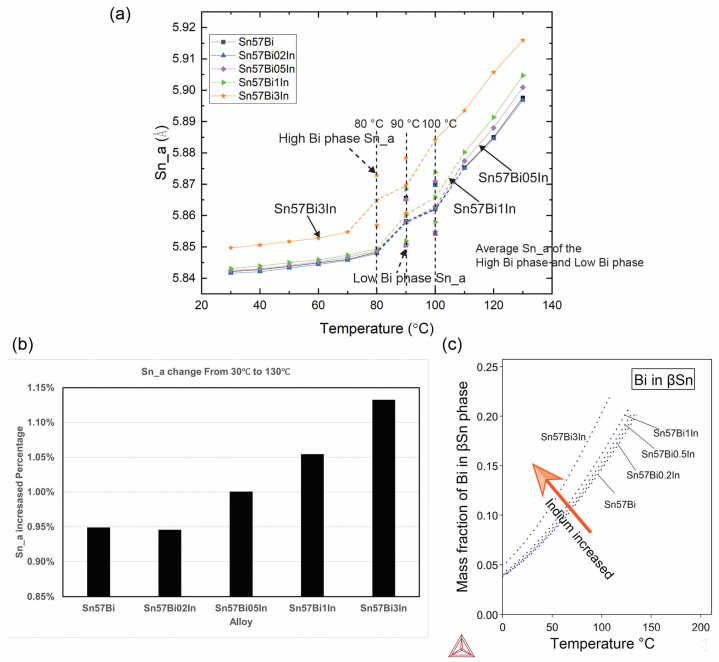
(**a**) Lattice parameter Sn–a vs. temperature for Sn–57Bi–xIn (x = 0, 0.2, 0.5, 1, and 3 wt%), (**b**) Sn–a changed percentage from 30 °C to 130 °C, and (**c**) mass fraction of Bi in βSn phase for Sn-57Bi–xIn (x = 0, 0.2, 0.5, 1, and 3 wt%), Thermo-calc.

Following Rietveld refinement of the PXRD patterns, with the quality of fit ensured by controlling the *R*_WP_ to below 10, the lattice parameters of the βSn phase were accurately determined. Figure 7 shows the lattice parameter Sn–a for the βSn phase during the heating process from 30 °C to 130 °C (lattice parameter Sn–c is presented in Figure S3). As depicted in Figure 7, the data points corresponding to the same temperature, where three distinct values are present, indicate the occurrence of peak splitting, as previously observed in Figure 6. The central data point of the triplet represents the average lattice parameter of the high Bi content phase and low Bi content phase (these averaged values are connected using dashed lines), signifying an intermediary state between high and low Bi dissolution within the βSn phase.

To accurately model the split peaks observed during the heating process, a dual-phase approach was employed during the Rietveld refinement. This method incorporates two distinct βSn phases to represent the varying degrees of Bi dissolution. Figure S4 exhibits a representative Rietveld refinement of the synchrotron PXRD pattern for Sn–57Bi at 100 °C. The refinement, which integrates these two βSn phases, shows an excellent fit to the experimental PXRD pattern.

The lattice parameter of the βSn phase is observed to increase with temperature; however, this increase is not linear, corroborating the findings from our previous research that highlight the significant role of Bi dissolution in lattice parameter expansion during temperature variations [17,24,25]. At a certain temperature, the rate of increase in the lattice parameter accelerates, this might be due to the Bi solubility increasing or the Bi atoms becoming more active and the dissolution speed increasing. This happens at around 80 °C for Sn–57Bi–3In and 90 °C for the other alloys. From the cooling curve analysis, it is found that BiIn formed around 90 °C and from the Thermal-Calc results in Figure S2c, the BiIn formed around 75 °C. Therefore, the BiIn is likely dissolved during heating at around 80 °C and this will contribute to increase the βSn’s lattice parameters.

The introduction of In leads to an enlargement of the βSn lattice. To quantitatively assess the dissolution of Bi into the βSn phase during heating, the change in percentage in terms of the lattice parameter, denoted Sn–a, from 30 °C to 130 °C, has been calculated and is depicted in Figure 7b. A comparison can be made given the identical temperature profile and experimental conditions applied to all the samples and considering that the alloys were stabilised post-casting in the laboratory for over a month. Figure S2 and the EDS results in Figure 2 indicate, after solidification, that the In is dissolved or forms the BiIn phase for the Sn–57Bi–3In alloy. In both scenarios, the In should not influence the lattice parameter during heating. Therefore, it can be inferred that the variations in Sn–a predominantly reflect the dissolution of Bi, absent other influencing factors.

Figure 7 billustrates the increase in the lattice parameter Sn–a at 130 °C, compared to 30 °C for the five alloys. The Sn–57Bi–0.5In, Sn–57Bi–1In, and Sn–57Bi–3In samples exhibit a more significant increase in Sn–a compared to the alloy without the In addition, whereas Sn–57Bi–0.2In mirrors the behaviour of the Sn–57Bi sample. This indicates that the In addition enhances the dissolution of Bi during the heating process in terms of the Sn–Bi system.

The Thermo-Calc simulations were employed to further investigate the dissolution behaviour of Bi during the βSn phase for varying concentrations of In, as shown in Figure 7c.The calculated mass fraction of Bi in the βSn phase, as a function of temperature, reveals an increase with the In addition, signifying a greater solubility of Bi. Moreover, this increase in mass fraction is accentuated with rising temperatures, a divergence that aligns with the experimental observations from the lattice parameter analysis. For example, at 30 °C the solubility of Bi in the βSn phase increased from about 6.1% to 8% after adding 3% In and at 100 °C the solubility of Bi in the βSn phase increased from about 14.6% to 20.3%. Thus, it is confirmed that the addition of In indeed promotes the dissolution of Bi within the βSn phase.

#### 3.3.4. PXRD Patterns during Cooling

The examination of the Sn–57Bi–xIn (where x = 0, 0.2, 0.5, 1, and 3 wt%) alloys during the heating phase has revealed the influence of In on Bi dissolution within the βSn phase as the temperature increases. However, solder alloys typically undergo both heating and cooling, making it crucial to understand the mechanisms during the cooling process to assess their reliability. Like the heating phase, the changes in the Sn peaks during the cooling process provide insights into the dissolution and precipitation dynamics of Bi. Consequently, the Sn (200) peak is also the focal point for comparative analysis during cooling.

**Figure 8:**
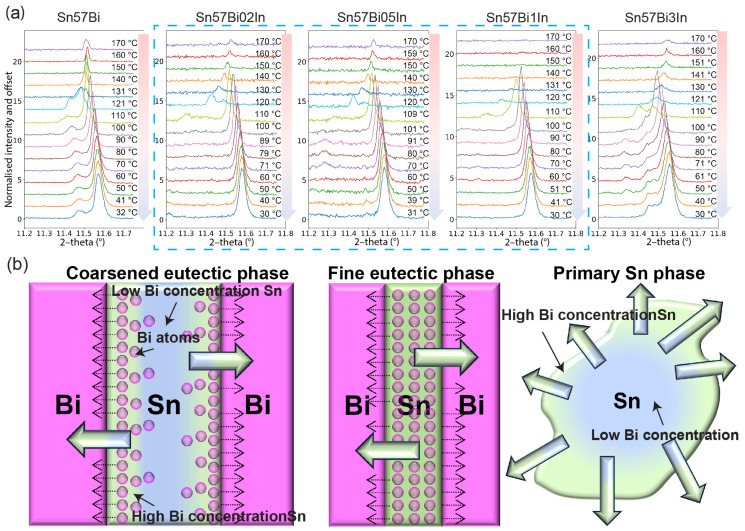
(**a**) Normalised Sn (200) peak during cooling for Sn–57Bi–xIn (x = 0, 0.2, 0.5, 1, and 3 wt%). (**b**) Schematic of the Bi concentration within the Sn phase.

Figure 8a presents the normalised Sn (200) peak during cooling, from a fully molten state at 170 °C to approximately room temperature at 30 °C. At 170 °C, given that the alloys are above their liquidus temperature, only a minor presence of the unmelted Sn phase is detectable. As the temperature descends towards the solidus, the Sn peaks intensify due to the solidification of the alloy. The subsequent rightward peak shift, the opposite to that observed during heating, indicates a reduction in the lattice parameter owing to the temperature decrease and Bi precipitation from the βSn phase.

For the Sn–57Bi alloy, the cooling process reveals peak splitting, suggesting a non-uniform Bi concentration within the βSn phase. The introduction of In, however, appears to homogenise the Bi distribution during cooling, as evidenced by the absence of peak splitting in the Sn–57Bi–0.2In, Sn–57Bi–0.5In, and Sn–57Bi–1In alloys. Yet, for Sn–57Bi–3In, peak splitting becomes pronounced. From our previous discussions, In can promote the dissolution of Bi during the βSn phase; it also has a facilitative effect during the precipitation process, making the distribution of Bi more homogeneous, compared with the Sn–57Bi alloy. However, for the Sn–57Bi–3In alloy, Figure 2 shows that the microstructure of the alloy is much coarser. It means the Sn dendrites are broadened compared with the other alloys, increasing the diffusion distance of the Bi. For coarse Sn dendrites and the primary βSn phase, the gradient of the Bi concentration forms, resulting in a large deviation in the lattice parameters and peak splitting, as shown in the schematics in Figure 8b. 

Combining the analysis during the heating process, it can be deducted that In additions influence the homogeneity of Sn–Bi-based solder alloys during thermal cycling. Depending on the application’s temperature range, the homogeneous temperature range during the heating process would be decreased by the In concentration; however, the homogeneity would be much improved by an additional 0.2 wt% to 1 wt% In. Therefore, selecting an appropriate In concentration is crucial for enhancing the reliability of the solder alloy. Based on the results in this article, it is suggested that the addition of 0.2 wt% to 1 wt% In would compromise both the heating and cooling process during thermal cycling, in terms of improving the stability of the solder alloy.

### 3.4. Atomistic Simulations

DFT calculations were employed to delve into the mechanistic aspects of In’s influence on Bi dissolution during the βSn phase. A series of models representing various compositions of Bi and In were constructed, as summarised in Table 1. The concentration of In in the Sn matrix ranged from 0 wt% to approximately 1.5 wt% to simulate the increase in In concentration in the alloy. The simulated Bi concentration ranged from 4.05 wt% to 10.51 wt%, a subset of the Bi concentration during the βSn phase of about 5% to over 20 wt%, as depicted in Figure 7c. Therefore, for Sn57BixIn alloys, the model’s concentration of Bi is in the range of the dissolution amount of Bi during the heating process and cooling process. Therefore, this model can simulate the conditions in terms of the different concentrations of Bi and In during the βSn phase, related to the experimental results.

All the DFT were derived from a 4 × 4 × 8 supercell of the tetragonal β-Sn unit cell, as depicted in Figure S5a. Given that In was observed to dissolve completely into the β-Sn phase, maintaining the tetragonal structure, we posited that In atoms substitute Sn atoms within the lattice. Therefore, during the concentration alteration process, Bi atoms are modelled to substitute Sn atoms, and vice versa, upon precipitation.

The models selected for the DFT calculations adhere to the criterion that the resultant structure post-calculation remains tetragonal, consistent with the PXRD observations. Table 1 details the specific atom counts and weight percentages for each element within the models.

**Figure 9:**
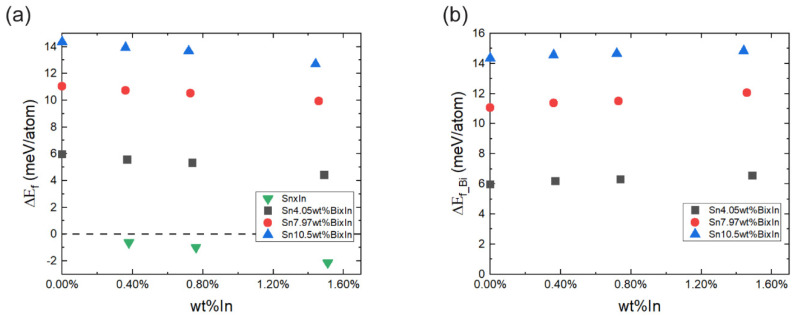
(**a**) Energy of formation for the dissolution of Bi and In, *ΔE_f_*, vs. In in the Sn–Bi–In alloy and (**b**) energy of formation for the dissolution of Bi, *ΔE_f_Bi_*, vs. In concentrations in the Sn–Bi–In alloy.

The DFT calculations were performed to ascertain the dissolution behaviour of In during the βSn phase. Models designated as Sn–xIn were generated by replacing two, four, and eight Sn atoms with In in a 512-atom Sn supercell, resulting in compositions of Sn510In2, Sn508In4, and Sn504In8, corresponding to 0.38 wt%, 0.76 wt%, and 1.51 wt% In, respectively (see Figure S5b–d). The formation energies, *ΔE_f_*, depicted as green points in Figure 9a, exhibit negative values for all In concentrations, indicating that the addition of In stabilizes the βSn phase more than pure Sn. A further decrease in the formation energy, *ΔE_f_*, with increasing In content, suggests a preference for In to dissolve into the Sn matrix.

To investigate the effect of In on Bi dissolution, DFT models Sn_512-y-z_Bi_y_In_z_, where y = 12, 24, and 32, and z = 0, 2, 4, and 8, were developed. These models, listed in Table 1 and visualised in Figure S6, represent varied Bi and In concentrations within the Sn–Bi–In system. As shown in Figure 9a, *ΔE_f_* increases with the Bi concentration, implying greater energy requirements for Bi dissolution into the Sn phase. This correlates with the experimental observation that Bi dissolves into the Sn phase during the heating process when sufficient energy for atom substitution is available. Also, *ΔE_f_* decreases as the concentration of In increases, suggesting that the presence of In in the Sn matrix stabilizes the structure, providing a viable explanation for the higher Bi solubility in Sn with the addition of In in the same thermal conditions.

Bi mobility, driven by temperature fluctuations, involves diffusion into and out of the Sn lattice during thermal cycles. The energy of formation associated with the Bi concentration, *ΔE_f_Bi_*, is given by Equation (2).

*ΔE_f_Bi_* = *ΔE_f_* [Sn_512-y-z_Bi_y_In_z_] – *ΔE_f_* [Sn_512-z_In_z_] (2)

where *ΔE_f_* [Sn_512-y-z_Bi_y_In_z_] and *ΔE_f_* [Sn_512-z_In_z_] are the formation energies for the Sn_512-y-z_Bi_y_In_z_ and the Bi-free Sn_512-z_In_z_ at the same In concentration, respectively.

Figure 9b illustrates that *ΔE_f_Bi_* maintains a relatively consistent, yet marginally increasing, trend with the In concentration, across all Bi levels. This indicates that the incorporation of In slightly elevates the energy barrier for Bi dissolution and precipitation.

In the micro-scale environment of electronic devices, where the thermal energy supply is substantial relative to the size of the solder, this minor difference in *ΔE_f_Bi_* is unlikely to significantly affect the dissolution and precipitation dynamics of Bi. Consistent with the experimental setup, where temperature ramping rates are controlled, the energy available for these processes can be considered effectively unlimited. Therefore, the slight increase in *ΔE_f_Bi_* due to the In addition does not substantially hinder Bi’s dissolution and precipitation. From the analysis above, the solubility of Bi in terms of Sn is increased by the introduction of In into the alloy, which promotes the dissolution or precipitation of Bi compared to the In-free alloy, in the same thermal conditions.

## 4. Conclusions

In conclusion, our comprehensive investigation into the Sn–57Bi–xIn alloy system has revealed the nuanced influence of In on both the microstructural evolution and thermal behaviour of these alloys. The conclusions can be summarised as follows:

(1) The addition of In into the Sn–Bi alloy system influences the morphology of the eutectic structure. Specifically, In enhances the refinement of the eutectic phase when present in concentrations of 1 wt% and below, whereas at a higher content of 3 wt%, it induces a coarsening effect;
(2) The observed trends in terms of the recalescence with varying In concentrations, coupled with microstructural observations, point to In’s bifunctional influence: it first refines and then coarsens the eutectic structure as its content increases. These findings highlight In’s potential as a microstructure modifier in solder alloys;
(3) In situ synchrotron PXRD analysis has yielded crucial insights into how In influences the dynamics of Bi dissolution and precipitation within the Sn matrix of Sn–Bi alloys. The findings demonstrate that In changes the homogeneous status during the Sn phase. During the heating process, the splitting temperature and merging temperature, which present an inhomogeneous status and a homogeneous status, respectively, decrease with the In concentration. Furthermore, the addition of In is confirmed to facilitate the dissolution of Bi into the Sn phase. In addition, there is an alloy-dependent temperature above which the dissolution speed of Bi into Sn increases significantly, and additions of In lower this temperature;
(4) During the cooling phase, In appears to contribute to a more uniform distribution of Bi within Sn–Bi alloys. However, the precipitation behaviour is also dependent on the microstructure: an excessively coarse structure, characterised by widened Sn dendrites, can lead to non-uniformity, as observed in the Sn–57Bi–3In alloy. This indicates that while In acts as a homogenising agent, its effectiveness is contingent upon the microstructural context, underlining the complex interplay between alloy composition, structure, and thermal behaviour. Therefore, selecting an appropriate In concentration to optimise the decrease in the initial inhomogeneous temperature and achieve the benefit of homogeneous cooling after adding In is essential for improving the solder alloy’s stability. Based on the results presented in this article, it is recommended that the addition of 0.2 wt% to 1 wt% In is used to optimise both the heating and cooling processes during thermal cycling;
(5) The DFT calculations further revealed the mechanism behind In’s effect, demonstrating that In stabilises the βSn phase and promotes more Bi dissolution in a given thermal condition. Despite a slight increase in the energy required for Bi dissolution and precipitation due to In, the abundance of thermal energy in micro-scale solder applications renders this increase negligible in practical experiments, resulting in an acceleration of the dissolution phenomena.

Based on the summarised results, the influence of In on the morphology of the Sn–Bi–In alloy has been revealed, showing that In can refine or coarsen the microstructure depending on its concentration. Additionally, the PXRD and DFT calculations indicate that In promotes the homogeneous dissolution and precipitation of Bi within the alloy. The optimal In concentration for enhancing thermal behaviour is between 0.2 wt% and 1 wt%.

In the broader context of solder alloy development for electronic devices, our findings reveal the mechanism behind the role of In in enhancing the thermal cycling reliability of Sn–Bi-based solders. By making Bi dissolution and precipitation more homogeneous, In incorporation could lead to solders with improved mechanical properties and longevity under thermal stresses. The mechanism could be applied to the future design of Sn–Bi solder alloys to meet industry requirements. Additionally, this work provides a method to investigate the effects of ternary elements on thermally sensitive, low-temperature solder alloys, such as Sn–Bi-based alloys and Sn–In-based alloys, which are essential for the electronics manufacturing industries.

## Figures and Tables

**Table 1:** The details of the DFT calculation models.

Models	Atoms: Number of Elements			Weight Percent of Elements	
**Sn–xIn**	Sn	Bi	In	wt%Bi	wt%In
Figure S5a	512	0	0	0.00%	0.00%
Figure S5b	510	0	2	0.00%	0.38%
Figure S5c	508	0	4	0.00%	0.76%
Figure S5d	504	0	8	0.00%	1.51%
**Sn–12Bi–xIn**	Sn	Bi	In	wt%Bi	wt%In
Figure S6a	500	12	0	4.05%	0.00%
Figure S6b	498	12	2	4.05%	0.37%
Figure S6c	496	12	4	4.05%	0.74%
Figure S6d	492	12	8	4.06%	1.49%
**Sn–24Bi–xIn**	Sn	Bi	In	wt%Bi	wt%In
Figure S6e	488	24	0	7.97%	0.00%
Figure S6f	486	24	2	7.97%	0.36%
Figure S6g	484	24	4	7.97%	0.73%
Figure S6h	480	24	8	7.97%	1.46%
**Sn–32Bi–xIn**	Sn	Bi	In	wt%Bi	wt%In
Figure S6i	480	32	0	10.50%	0.00%
Figure S6j	478	32	2	10.50%	0.36%
Figure S6k	476	32	4	10.51%	0.72%
Figure S6l	472	32	8	10.51%	1.44%

## Data Availability

All data supporting the results of this study are included within the article.

## Supplementary Materials

The following supporting information can be downloaded at: https://www.mdpi.com/article/10.3390/ma17174372/s1, Figure S1: Area percentage of primary Sn dendrites in the Sn-57Bi-xIn (x = 0,0.2, 0.5, 1 and 3 wt%) alloys; Figure S2: Solidification process of (a) Sn57Bi0.5In (b) Sn57Bi1In and (c) Sn57Bi3In derived by Thermo-Calc; Figure S3: Lattice parameter Sn_c vs temperature for Sn-57Bi-xIn (x = 0, 0.2, 0.5, 1 and 3 wt%); Figure S4: Rietveld refinement of the synchrotron PXRD pattern for Sn57Bi at 100 °C. (Enlarged area is the split Sn peaks; Figure S5: DFT simulation models of (a) Sn512, (b) Sn510In2 (0.38 wt% In), (c) Sn508In4 (0.76 wt% In) and (d) Sn504In8 (1.51 wt% In); Figure S6: DFT simulation models of (a) Sn500Bi12 (4.05wt%Bi), (b) Sn498Bi12In2, (c) Sn496Bi12In4, (d) Sn492Bi12In8, (e) Sn488Bi24 (7.97wt%Bi), (f) Sn486Bi24In2, (g) Sn484Bi24In4, (h) Sn480Bi24In8, (i) Sn480Bi32 (10.50wt%Bi), (j) Sn478Bi32In2, (k) Sn476Bi32In4 and (l) Sn472Bi32In8.
