# Supplementary material for: The Effect of In Concentration and Temperature on Dissolution and Precipitation in Sn–Bi Alloys
Source: Materials (Basel). 2024 Sep 4;17(17):4372. doi: 10.3390/ma17174372 (PMC11396625; doi:10.3390/ma17174372)
Supplement: Supplementary file 1 [file materials-17-04372-s001.zip › materials-3162702-supplementary.pdf]

materials

Supplementary Materials for

The effect of In concentration and temperature on dissolution and precipitation in Sn-Bi alloys

Qichao Hao[1], Xin F. Tan[1], Qinfen Gu[2], Stuart D. McDonald[1], Kazuhiro Nogita[1*]

[1] Nihon Superior Centre for the Manufacture of Electronic Materials, School of Mechanical and Mining Engineering, The University of Queensland, St. Lucia, QLD 4072, Australia
[2] Australian Synchrotron, ANSTO, Clayton, VIC 3168, Australia
* Correspondence: k.nogita@uq.edu.au; Tel.: +61 7 336 53919

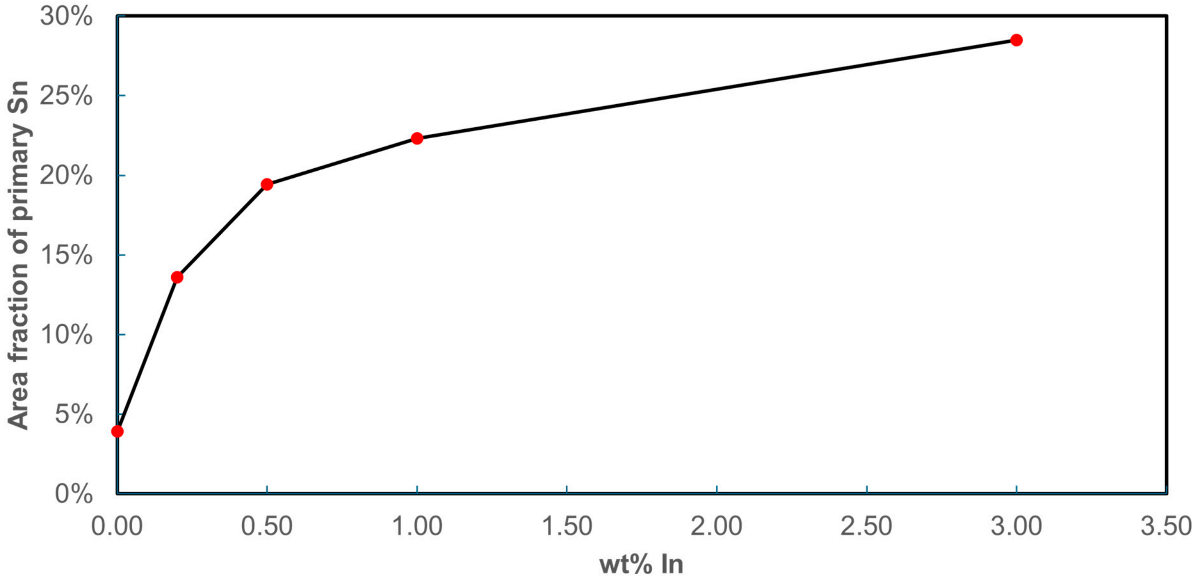

Figure S1. Area percentage of primary Sn dendrites in the Sn-57Bi-xIn (x = 0,0.2, 0.5, 1 and 3 wt%) alloys.

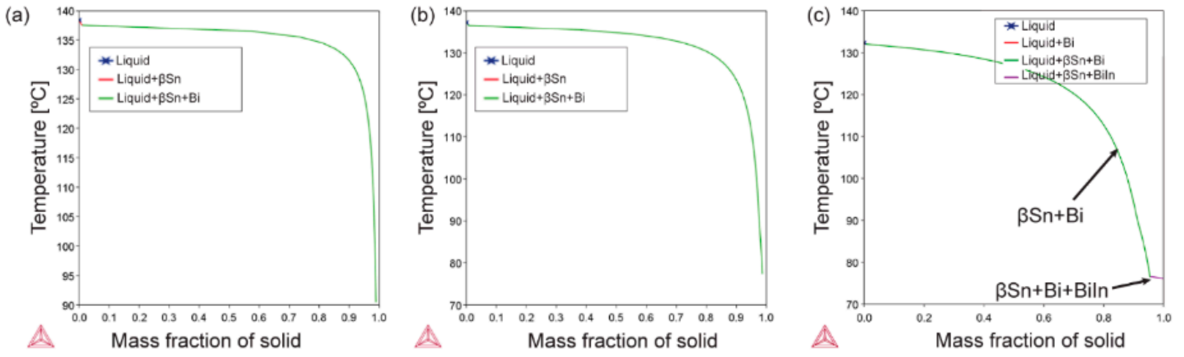

Figure S2. Solidification process of (a) Sn57Bi0.5In (b) Sn57Bi1In and (c) Sn57Bi3In derived by Thermo-Calc.

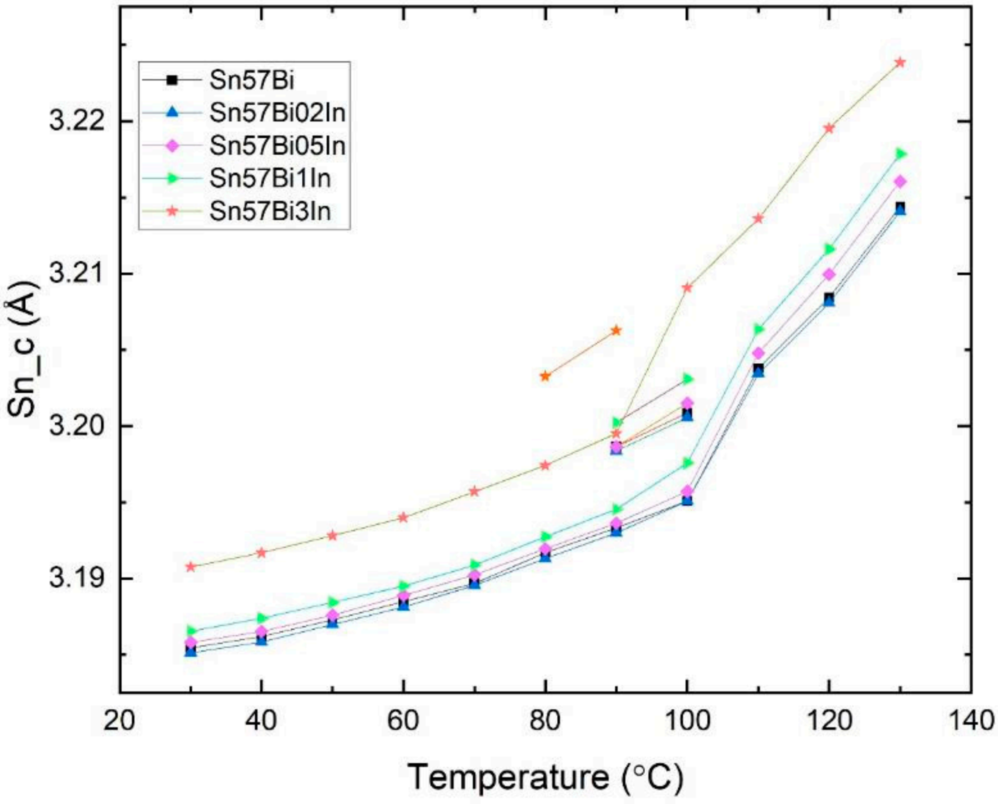

Figure S3. Lattice parameter Sn_c vs temperature for Sn-57Bi-xIn (x = 0, 0.2, 0.5, 1 and 3 wt%).

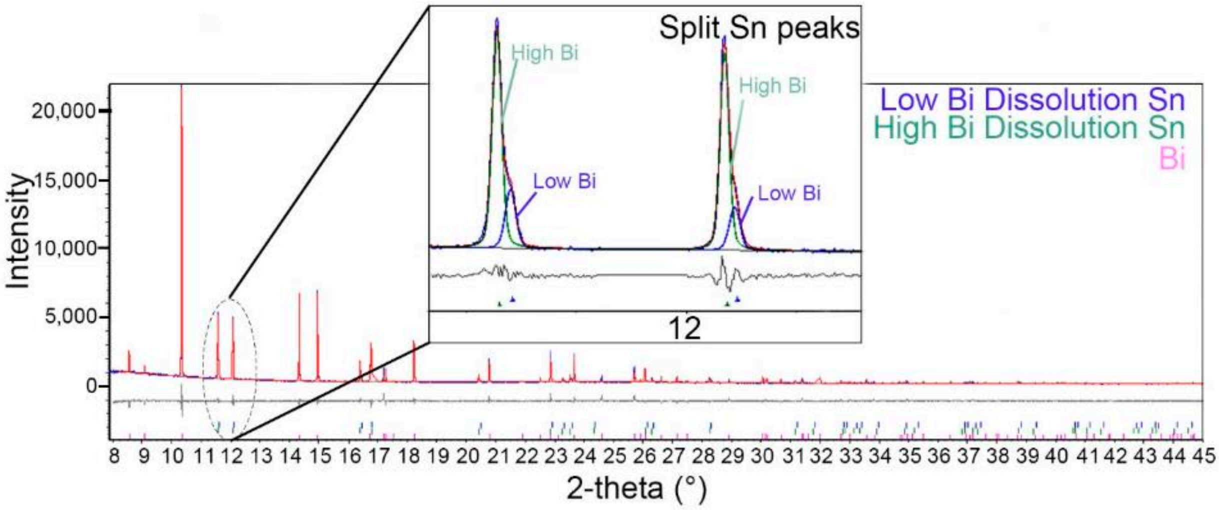

Figure S4. Rietveld refinement of the synchrotron PXRD pattern for Sn57Bi at 100 °C. (Enlarged area is the split Sn peaks.

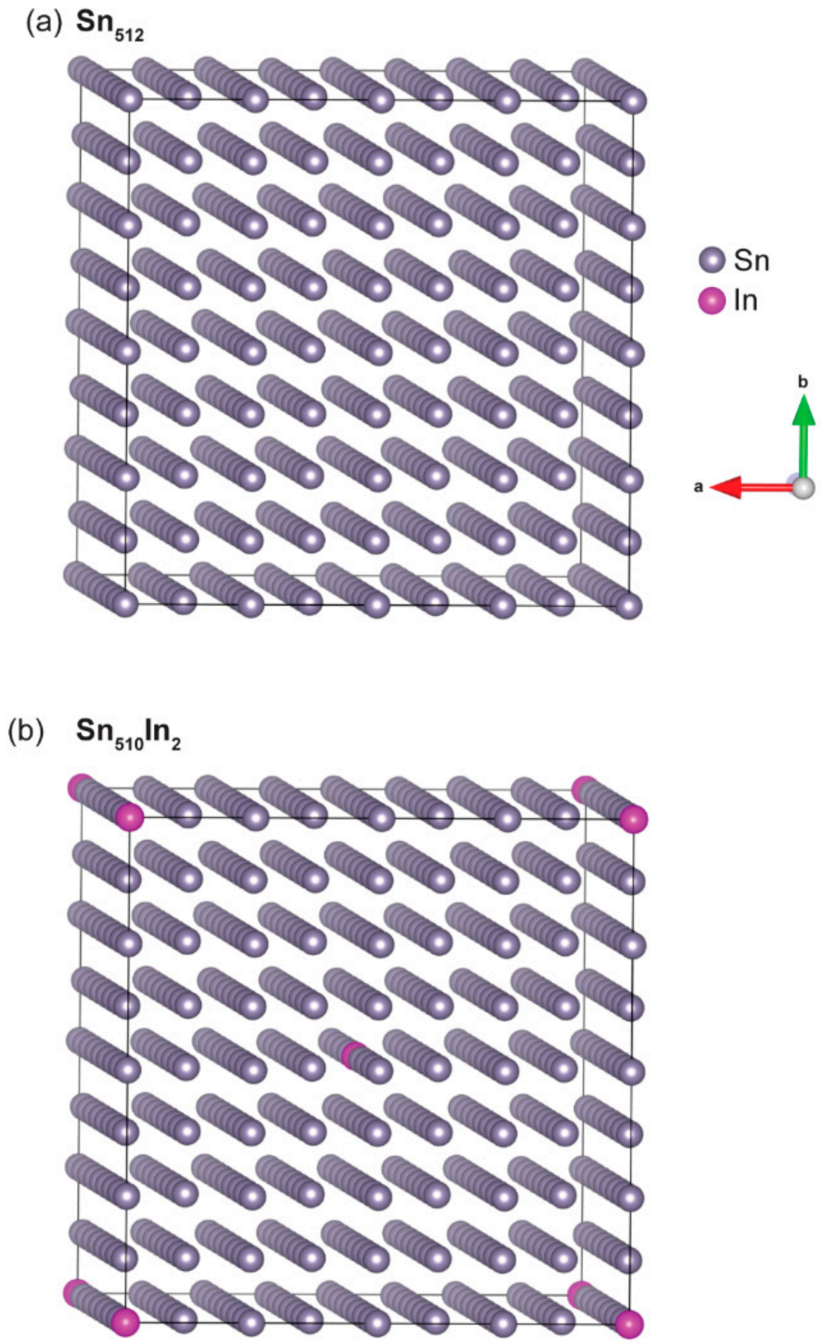

Figure S5. DFT simulation models of (a) Sn_{512}, (b) $Sn_{510}In_2$ (0.38 wt% In), (c) $Sn_{508}In_4$ (0.76 wt% In) and (d) $Sn_{504}In_8$ (1.51 wt% In).

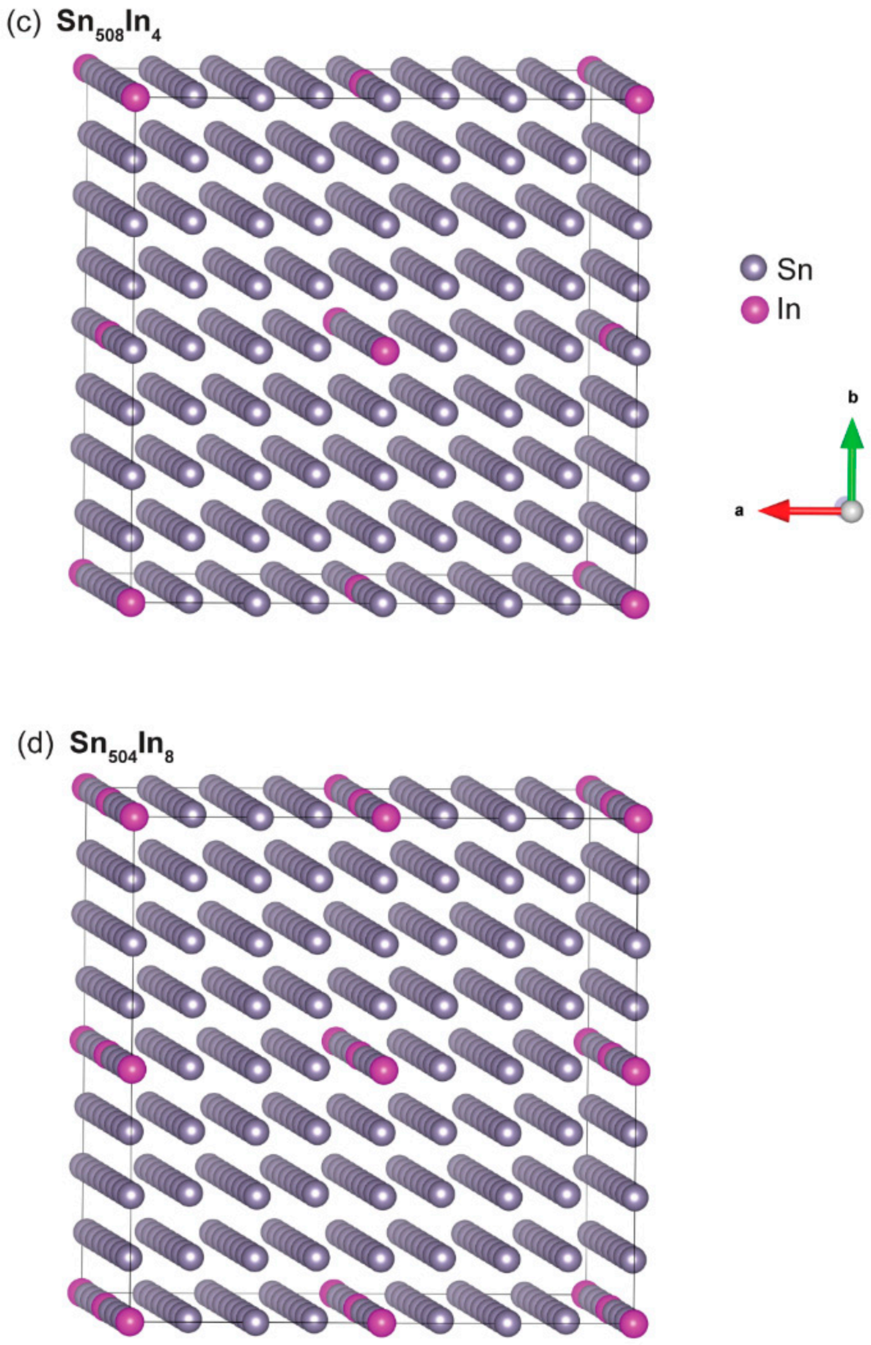

(Continued) Figure S5. DFT simulation models of (a) Sn_{512}, (b) $Sn_{510}In_2$ (0.38 wt% In), (c) $Sn_{508}In_4$ (0.76 wt% In) and (d) $Sn_{504}In_8$ (1.51 wt% In).

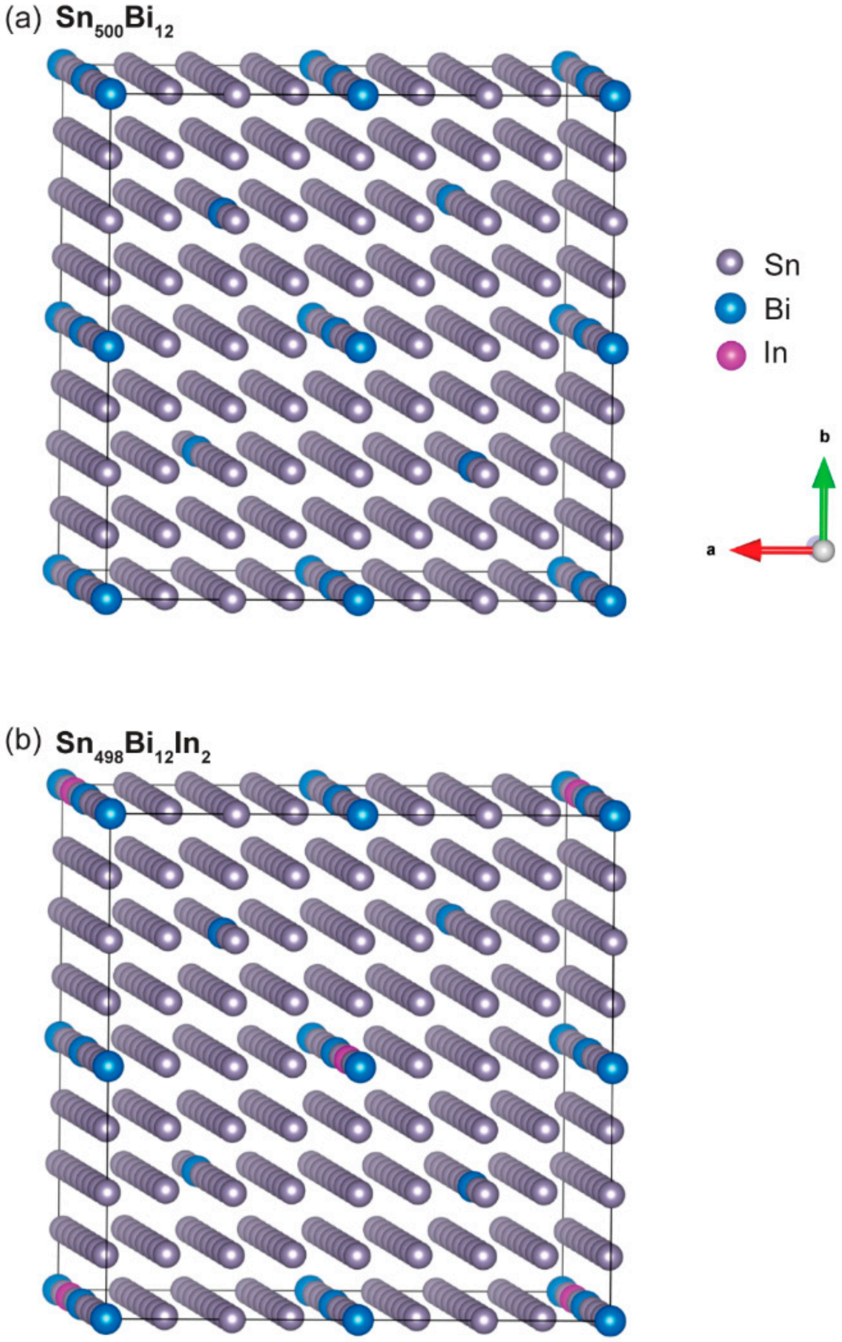

Figure S6. DFT simulation models of (a) $Sn_{500}Bi_{12}$ (4.05wt%Bi), (b) $Sn_{498}Bi_{12}In_2$, (c) $Sn_{496}Bi_{12}In_4$, (d) $Sn_{492}Bi_{12}In_8$, (e) $Sn_{488}Bi_{24}$ (7.97wt%Bi), (f) $Sn_{486}Bi_{24}In_2$, (g) $Sn_{484}Bi_{24}In_4$, (h) $Sn_{480}Bi_{24}In_8$, (i) $Sn_{480}Bi_{32}$ (10.50wt%Bi), (j) $Sn_{478}Bi_{32}In_2$, (k) $Sn_{476}Bi_{32}In_4$ and (l) $Sn_{472}Bi_{32}In_8$.

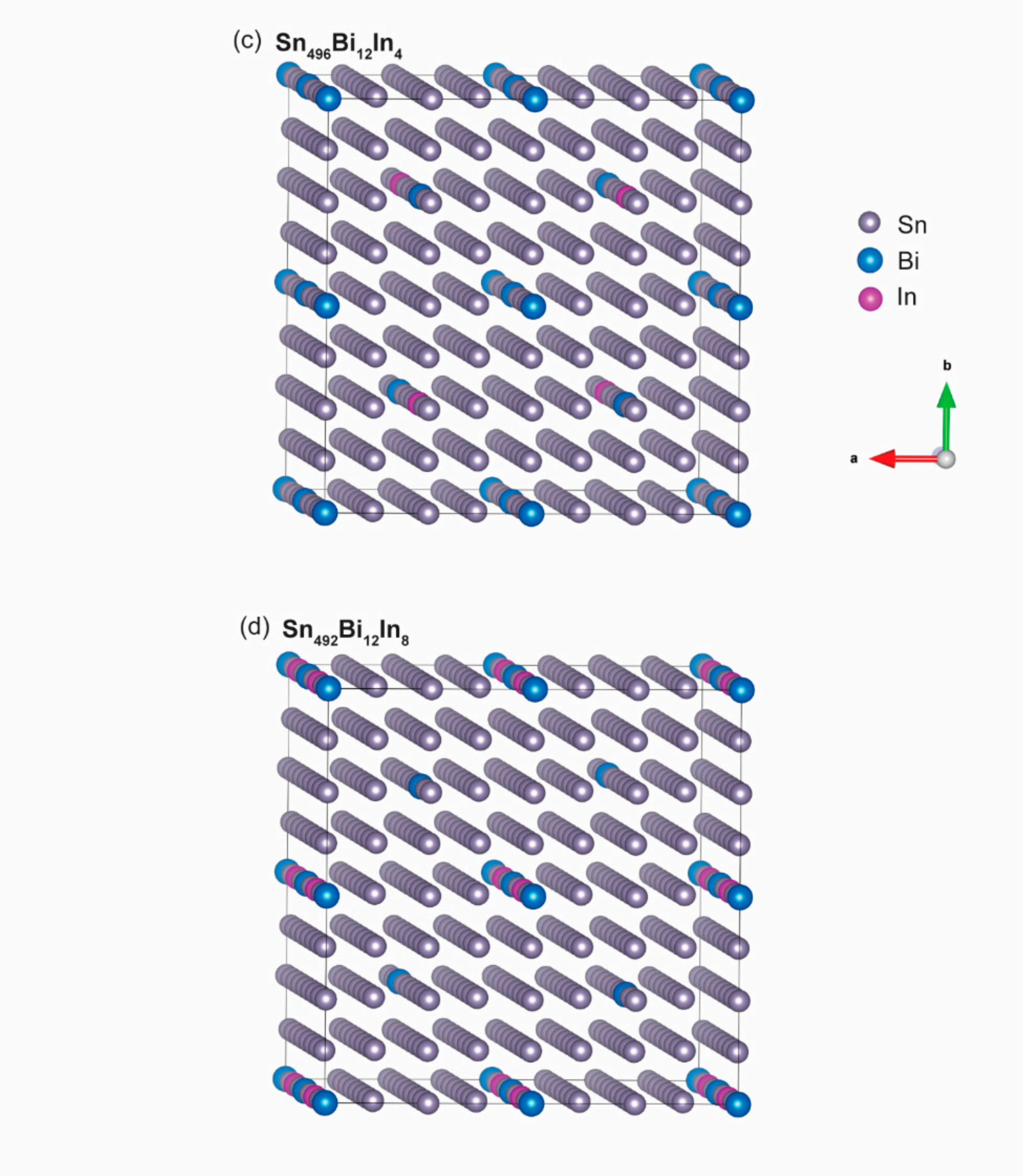

(Continued) Figure S6. DFT simulation models of (a) $Sn_{500}Bi_{12}$ (4.05wt%Bi), (b) $Sn_{498}Bi_{12}In_2$, (c) $Sn_{496}Bi_{12}In_4$, (d) $Sn_{492}Bi_{12}In_8$, (e) $Sn_{488}Bi_{24}$ (7.97wt%Bi), (f) $Sn_{486}Bi_{24}In_2$, (g) $Sn_{484}Bi_{24}In_4$, (h) $Sn_{480}Bi_{24}In_8$, (i) $Sn_{480}Bi_{32}$ (10.50wt%Bi), (j) $Sn_{478}Bi_{32}In_2$, (k) $Sn_{476}Bi_{32}In_4$ and (l) $Sn_{472}Bi_{32}In_8$.

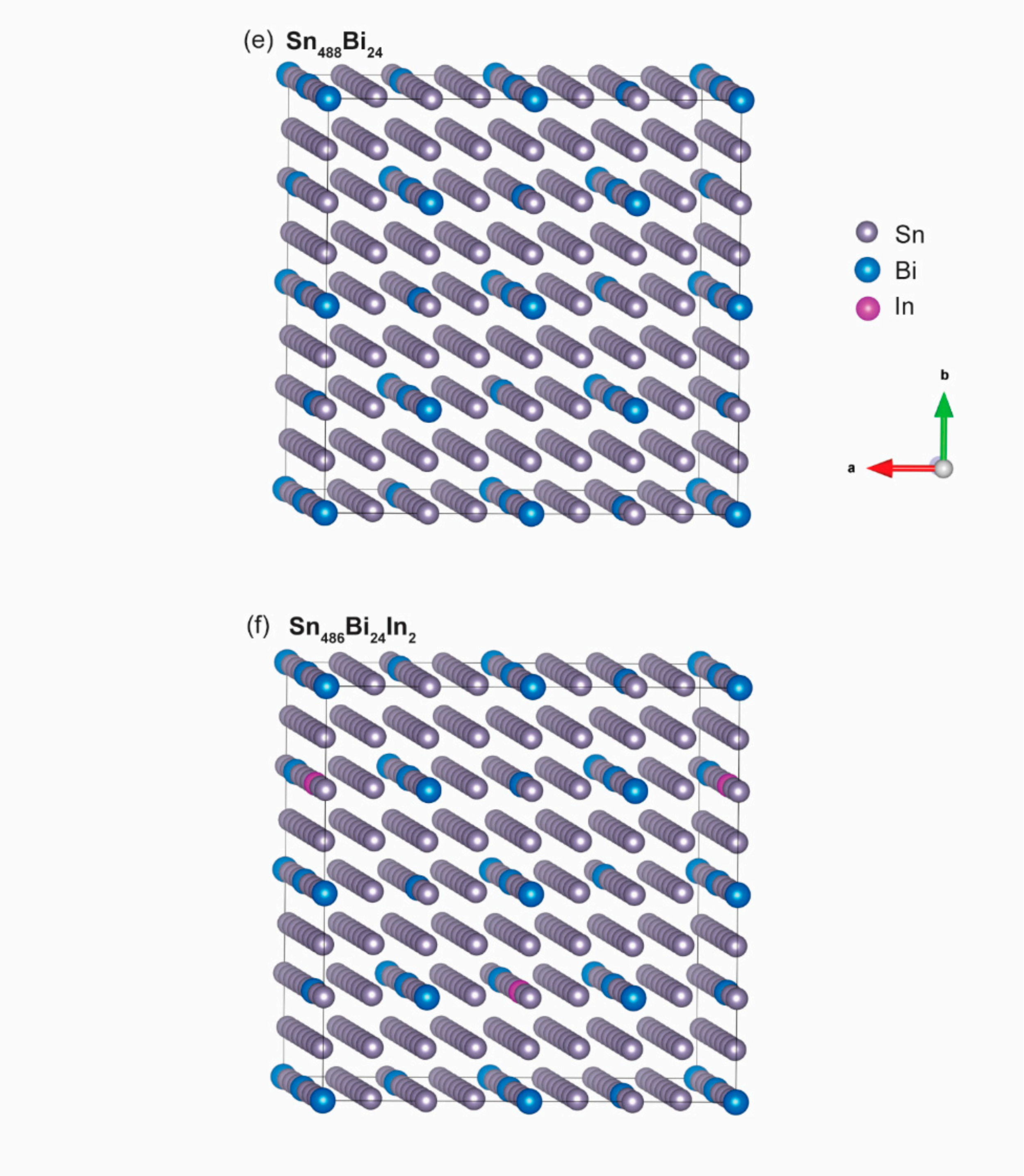

(Continued) Figure S6. DFT simulation models of (a) $Sn_{500}Bi_{12}$ (4.05wt%Bi), (b) $Sn_{498}Bi_{12}In_2$, (c) $Sn_{496}Bi_{12}In_4$, (d) $Sn_{492}Bi_{12}In_8$, (e) $Sn_{488}Bi_{24}$ (7.97wt%Bi), (f) $Sn_{486}Bi_{24}In_2$, (g) $Sn_{484}Bi_{24}In_4$, (h) $Sn_{480}Bi_{24}In_8$, (i) $Sn_{480}Bi_{32}$ (10.50wt%Bi), (j) $Sn_{478}Bi_{32}In_2$, (k) $Sn_{476}Bi_{32}In_4$ and (l) $Sn_{472}Bi_{32}In_8$.

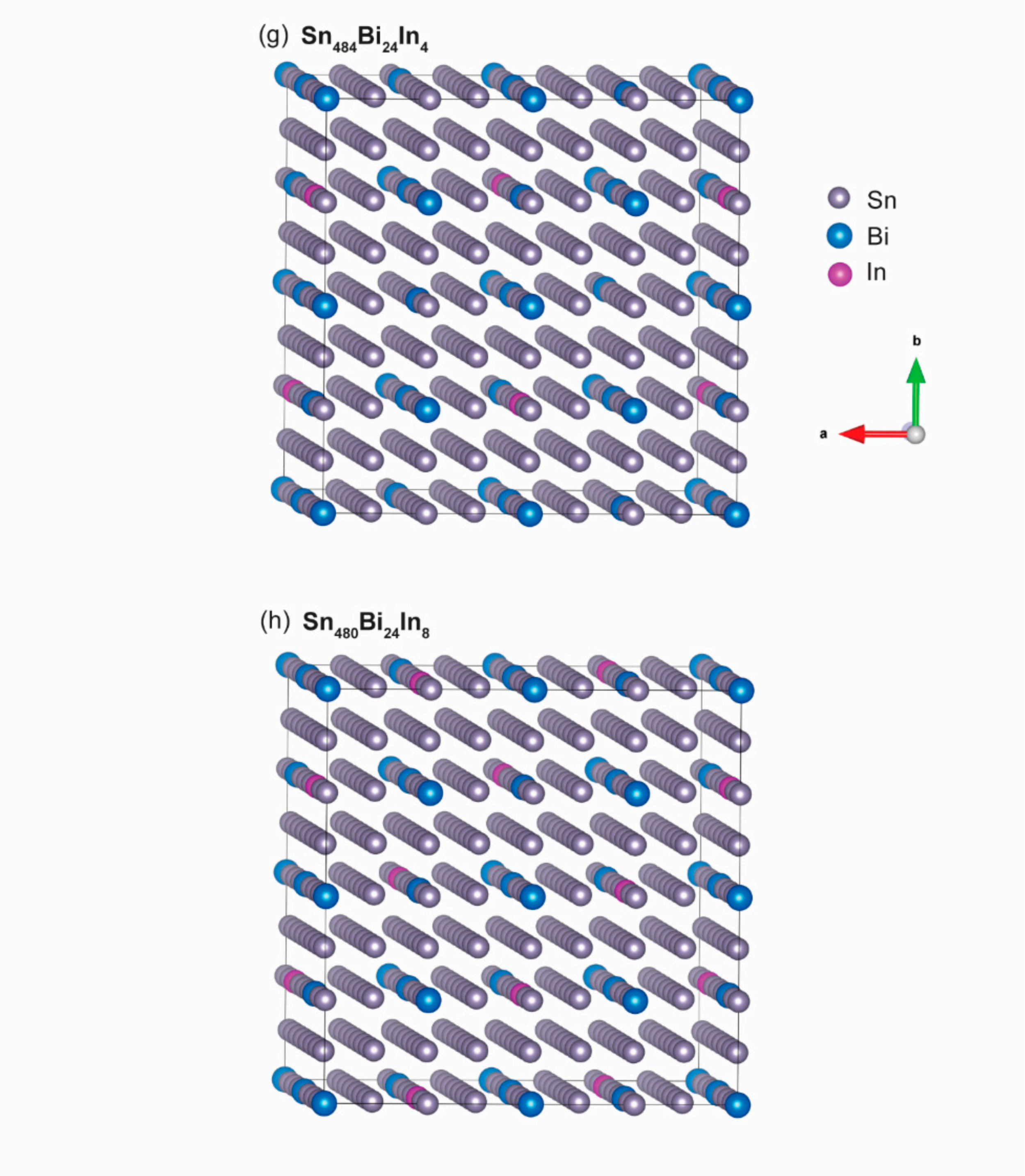

(Continued)

Figure S6. DFT simulation models of (a) $Sn_{500}Bi_{12}$ (4.05wt%Bi), (b) $Sn_{498}Bi_{12}In_2$, (c) $Sn_{496}Bi_{12}In_4$, (d) $Sn_{492}Bi_{12}In_8$, (e) $Sn_{488}Bi_{24}$ (7.97wt%Bi), (f) $Sn_{486}Bi_{24}In_2$, (g) $Sn_{484}Bi_{24}In_4$, (h) $Sn_{480}Bi_{24}In_8$, (i) $Sn_{480}Bi_{32}$ (10.50wt%Bi), (j) $Sn_{478}Bi_{32}In_2$, (k) $Sn_{476}Bi_{32}In_4$ and (l) $Sn_{472}Bi_{32}In_8$.

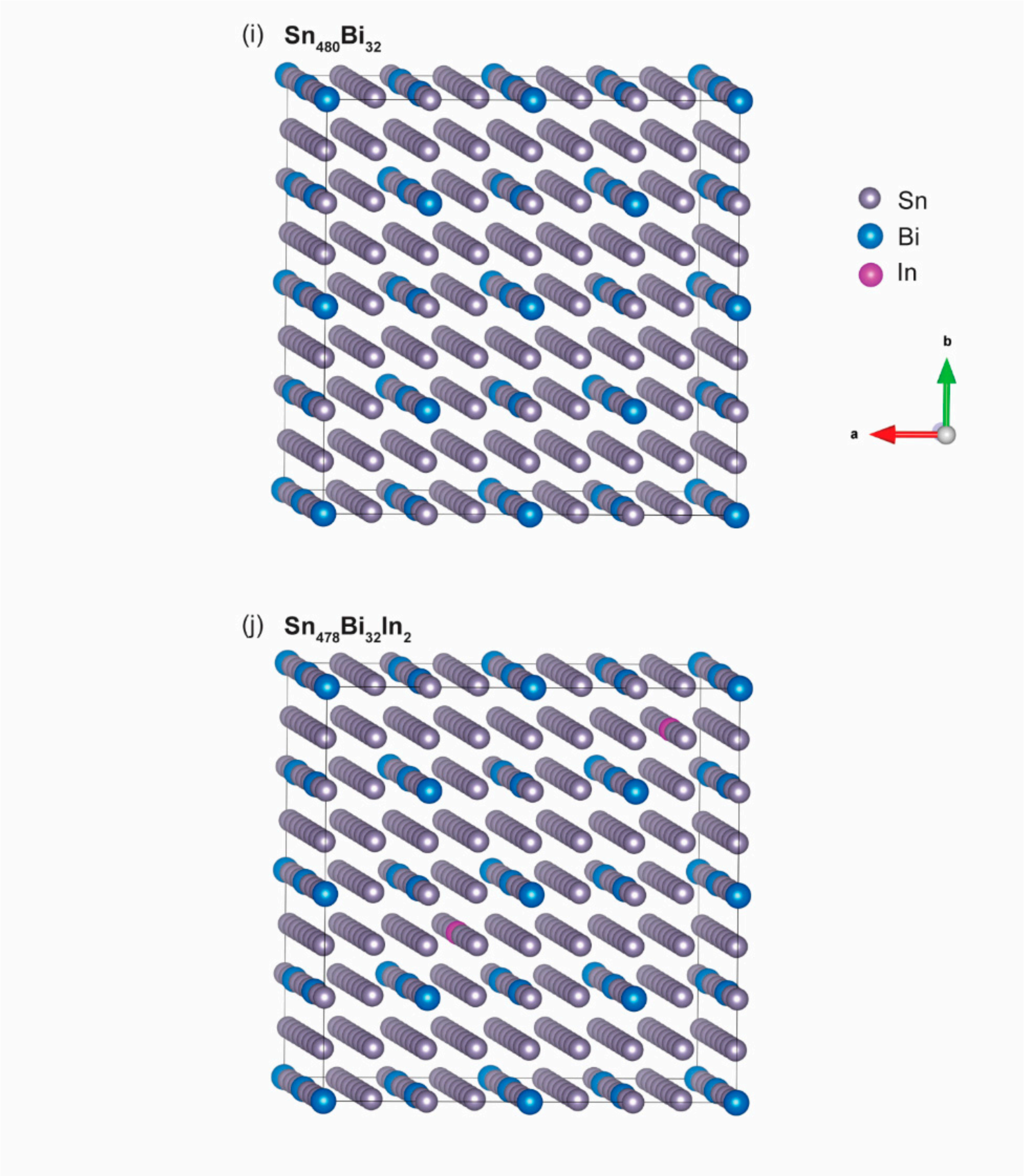

(Continued) Figure S6. DFT simulation models of (a) $Sn_{500}Bi_{12}$ (4.05wt%Bi), (b) $Sn_{498}Bi_{12}In_2$, (c) $Sn_{496}Bi_{12}In_4$, (d) $Sn_{492}Bi_{12}In_8$, (e) $Sn_{488}Bi_{24}$ (7.97wt%Bi), (f) $Sn_{486}Bi_{24}In_2$, (g) $Sn_{484}Bi_{24}In_4$, (h) $Sn_{480}Bi_{24}In_8$, (i) $Sn_{480}Bi_{32}$ (10.50wt%Bi), (j) $Sn_{478}Bi_{32}In_2$, (k) $Sn_{476}Bi_{32}In_4$ and (l) $Sn_{472}Bi_{32}In_8$.

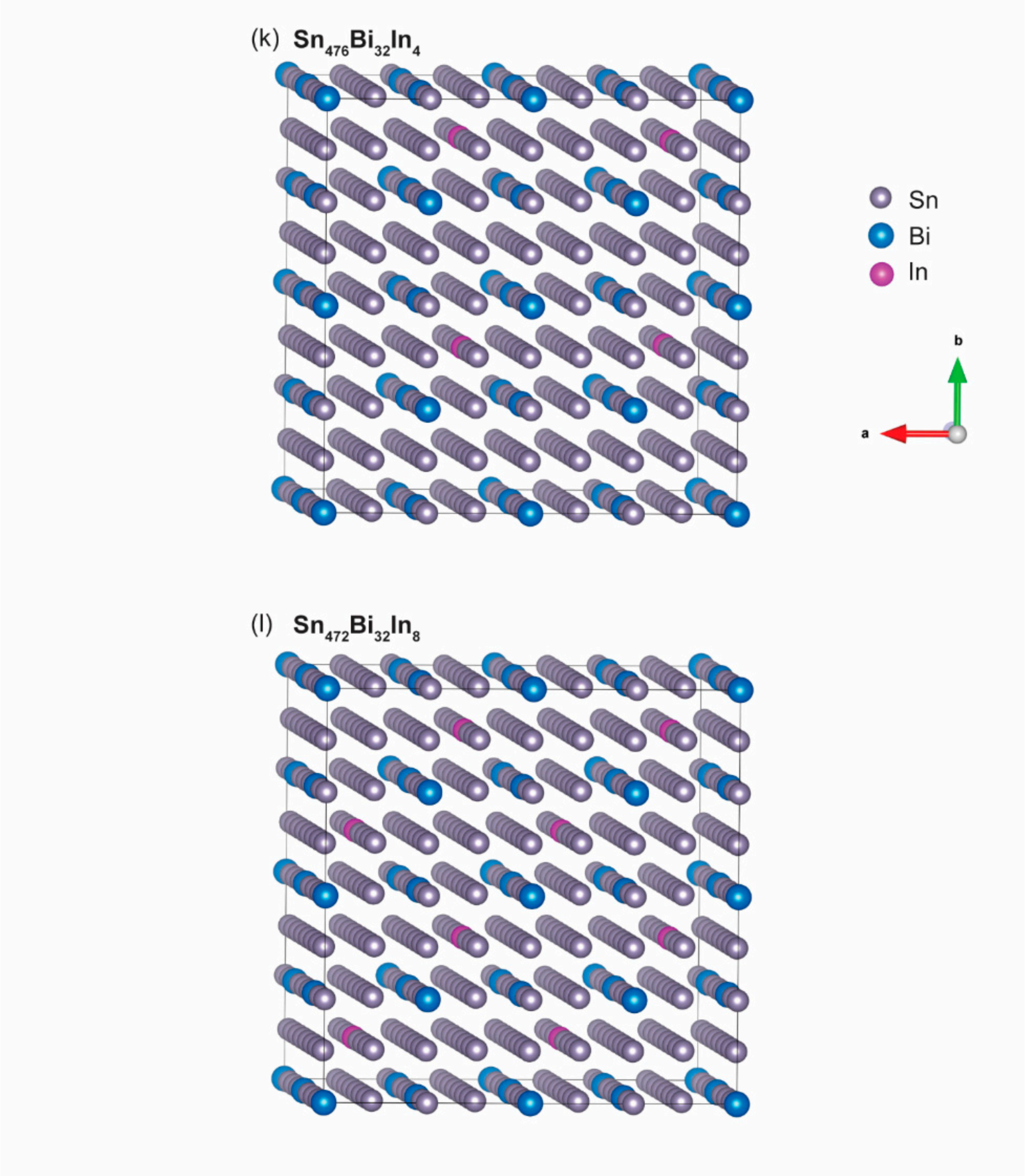

(Continued) Figure S6. DFT simulation models of (a) $Sn_{500}Bi_{12}$ (4.05wt%Bi), (b) $Sn_{498}Bi_{12}In_2$, (c) $Sn_{496}Bi_{12}In_4$, (d) $Sn_{492}Bi_{12}In_8$, (e) $Sn_{488}Bi_{24}$ (7.97wt%Bi), (f) $Sn_{486}Bi_{24}In_2$, (g) $Sn_{484}Bi_{24}In_4$, (h) $Sn_{480}Bi_{24}In_8$, (i) $Sn_{480}Bi_{32}$ (10.50wt%Bi), (j) $Sn_{478}Bi_{32}In_2$, (k) $Sn_{476}Bi_{32}In_4$ and (l) $Sn_{472}Bi_{32}In_8$.
